# Characterization of Endogenous Retroviral-like Particles Expressed from the *Spodoptera frugiperda* Sf9 Cell Line

**DOI:** 10.3390/v17020136

**Published:** 2025-01-21

**Authors:** Hailun Ma, Eunhae H. Bae, Pei-Ju Chin, Arifa S. Khan

**Affiliations:** 1 Division of Viral Products, Office of Vaccines Research and Review, Center for Biologics Evaluation and Research, U.S. Food and Drug Administration, Silver Spring, MD 20993, USA; hailun.ma@fda.hhs.gov (H.M.); pei-ju.chin@fda.hhs.gov (P.-J.C.); 2 Northwest Regional Campus, University of Arkansas for Medical Sciences, 1125 N. College Ave, Fayetteville, AR 72703, USA; eunhaebae@gmail.com

**Keywords:** *Spodoptera frugiperda*, Sf9 cell line, reverse transcriptase (RT), endogenous retroviral-like particles (RVLPs), extracellular vesicles (EVs), chemical induction, infectivity assay, transmission electron microscopy (TEM), cryo-electron microscopy (cryoEM), PCR-enhanced RT (PERT) assay

## Abstract

The *Spodoptera frugiperda* Sf9 insect cell line is used in the baculovirus expression vector system for the development of various viral vaccines and some gene therapy products. Early studies indicated that Sf9 cells produced a reverse transcriptase (RT) activity that was detected using a sensitive PCR-enhanced reverse transcriptase (PERT) assay. Since RT is generally associated with retrovirus particles, we undertook the investigation of the physical properties and infectious nature of the extracellular RT activity that was constitutively expressed from Sf9 cells or induced after the chemical treatment of the cells with drugs known to activate endogenous retroviruses. A density gradient analysis indicated that the peak RT activity corresponded to a low buoyant density of about 1.08 g/mL. Ultracentrifugation and size filtration of cell-free Sf9 supernatant indicated that different particle sizes were associated with the RT activity. This was confirmed by transmission electron microscopy and cryoEM, which revealed a diversity in particle size and type, including viral-like and extracellular vesicles. The treatment of Sf9 cells with 5-iodo-2′-deoxyuridine (IUdR) induced a 33-fold higher RT activity with a similar low buoyant density compared to untreated cells. Infectivity studies using various target cells (human A204, A549, MRC-5, and Raji, and African green monkey Vero cells) inoculated with cell-free supernatant from untreated and IUdR-treated Sf9 cells showed the absence of a replicating retrovirus by PERT-testing of cell-free supernatant during the 30 day-culturing period. Additionally, there was no evidence of virus entry by whole genome analysis of inoculated MRC-5 cells using high-throughput sequencing. This is the first study to identify extracellular retroviral-like particles in *Spodoptera*.

## 1. Introduction

Baculovirus-insect cell expression systems are a robust platform for large-scale, and rapid protein expression and have been used for the development of various investigational products and the manufacturing of several U.S. licensed human viral vaccines and therapeutics [1,2,3,4]. Since insects are phylogenetically very distant from humans, insect-based cell lines are generally considered to have less safety concerns related to adventitious viruses than mammalian cells for the manufacturing of biological products [for example, Sf9, Sf21, and High Five insect cell lines]. However, the detection of RT activity in the cell-free supernatant of Sf cells [5,6] raised potential safety concerns regarding the presence of retroviruses in the Sf9 cell substrate for manufacturing of biologics. A major scientific hurdle was the limited knowledge regarding endogenous retroelements in *Spodoptera*. To date, nine errantivirus sequences have been identified in the Sf9 cell genome by PCR amplification [7], and Sf-Maverick, R1 LINE, and errantivirus transposon sequences were reported in the Sf-RVN cells by bioinformatics analysis [8]. However, there have been no reports of retrovirus-like particles (RVLPs) in *Spodoptera frugiperda* or Sf cell lines. Insect endogenous retroviral elements have been characterized in other insect cell lines [9,10] and although they are generally repressed by PIWI-interacting RNAs [11,12], the presence of RVLPs has been reported in some species. In the case of *Drosophila*, intracytoplasmic A-type retrovirus-like toroidal particles of about 40 nm in diameter were found in *Drosophila melanogaster* cells and an RT activity with a density of 1.22 g/mL was reported by the sucrose gradient analysis of cell lysate [13]. Additionally, *gypsy* virus-like particles, spherical 50–75 nm in diameter with 1.22 g/mL density, were reported to be expressed extracellularly from *D. melanogaster* and *D. virilis* cell lines [14]. Virus-like particles with a diameter of 55–60 nm could be assembled from the baculovirus-expressed gag-like and pol-like ORFs of retrotransposon TED [15]. Furthermore, the ZAM retrotransposon in *D. melanogaster* can assemble virus particles with a diameter of about 45 nm in the posterior follicle cells of ovarian follicles [16].

Our laboratory previously developed a stepwise strategy for optimizing the chemical induction assay to investigate the presence of latent viruses, including endogenous retroviruses and oncogenic DNA viruses [17,18]. We applied this strategy to demonstrate the presence of inducible, endogenous retroviruses in the African green monkey Vero cell line with 5-azacytidine (AzaC) and 5-iodo-2′-deoxyuridine (IUdR) [19]. It was not known whether this chemical induction strategy could induce endogenous retroelements or retroviruses from insect cells. In this paper, we have characterized the RT activity that is constitutively produced from Sf9 cells and investigated the induction of endogenous retroviral particles from chemically treated Sf9 cells. Since Sf9 cells or clones derived from them are used for the manufacturing of biological products, we have evaluated the potential risk of the Sf-RT particles for human infection by conducting infectivity studies using selected cell lines with a known susceptibility for retroviruses, as well as to a broad range of other viruses [20,21,22,23].

## 2. Materials and Methods

Cell lines. The Sf9 cell line, which was derived from pupal ovarian tissue of *S. frugiperda*, was obtained from the American Type Culture Collection (ATCC, Manassas, VA, USA, catalogue number CRL-1711, lot no. 58078522, passage 16). The cells were grown as an adherent culture, as previously described [24] in Grace’s Supplemented Insect Medium (Invitrogen, Waltham, MA, USA, catalogue number 11605), supplemented with 10% fetal bovine serum (FBS, certified for insect cells, Hyclone, Logan, UT, USA, catalogue number SH30070.03; heat inactivated 56 °C for 30 min), 2 mM L-glutamine, 100 U of penicillin per mL and 100 µg of streptomycin per mL (Quality Biologicals Inc., Gaithersburg, MD, USA; catalogue number 120-095-721).

The Sf-rhabdovirus-negative Sf9-13F12 cell clone was isolated in our laboratory from Sf9 cells by the limiting dilution method [25] and grown, as described above, for Sf9 cells.

High Five Cells (BT1-TN-5B1-4; *Trichoplusi ni* ovarian cells, Invitrogen, catalogue number B855-02) were grown in Express Five SFM (serum-free medium, Invitrogen catalogue number 10486-025) as previously described [24].

Schneider’s Drosophila Line 2 [D. Mel. (2), SL2, *D. melanogaster* embryo cells, ATCC, catalogue number CRL-1963] were grown in Schneider’s Drosophila medium (Invitrogen catalogue number 21720-024) and supplemented with 10% heat-inactivated FBS (certified for insect cells) as previously described [24].

The target cell lines used for infectivity studies were obtained from the ATCC and included the following: A-204 (human rhabdomyosarcoma; ATCC catalogue number HTB-82), Raji (human B cell lymphoma; ATCC catalogue number CCL-86), MRC-5 (human fetal lung fibroblasts; ATCC catalogue number CCL-171), Vero (African green monkey kidney; ATCC catalogue number CCL-81), A549 (human lung carcinoma, ATCC catalogue number CCL-185). All cell lines were grown in a medium supplemented with 10% heat-inactivated FBS (56 °C for 30 min), 2 mM L-glutamine, 100 U of penicillin per mL, and 100 µg of streptomycin per mL: MRC-5 and Vero were grown in Eagle’s minimum essential medium (modified with Earle’s salts without L-glutamine (EMEM; Mediatech, Manassas, VA, USA; catalogue number 15-010-CV), 1x nonessential amino acids (MEM-NEAA, Quality Biological; catalogue number 116-078-721), and 1 mM sodium pyruvate (Quality Biological; catalogue number 116-079-060); A204 and Raji were grown in RPMI 1640 medium (Quality Biologicals, catalogue number 112-024-101) and 1x MEM-NEAA; and A549 was grown in Dulbecco’s modified Eagle’s MEM (DMEM; Invitrogen; catalogue number 119955).

Virus stock. Simian foamy virus type 1 (SFV-1) was obtained from the ATCC (Catalogue number VR 276) and a high-titer virus stock (10^5.5^ TCID_50_ per mL) was prepared by low passage in *Mus dunni* cells and tittered as previously described [21]. This was included as a positive control for retrovirus infection in the infectivity study.

PCR-enhanced RT (PERT) assay. Cell supernatants were filtered (0.45 μm, Corning, Corning, NY, USA; catalogue number 430314),and stored in aliquots at −80 °C in single-use tubes. The protocol used for the PERT assay was a two-step assay [21], which was based on the previously described method [26,27]. Briefly, the cell supernatant sample was prepared by diluting at 1:10 in NZ/DTT buffer per the standard protocol (to reduce interference due to serum in the medium), and in the case of direct testing of Sf9 supernatant, an additional 1:10 dilution was needed to reduce interference due to the insect cell culture medium. A standard curve of ten-fold serial dilutions of purified HIV-1 RT enzyme (Worthington Biochemical Corp, Lakewood, NJ, USA; Catalogue number RTHIV) was generated for each assay based on the enzymatic activity provided for the lot used in the assay. A medium or water was used as the negative control. Each sample was tested in duplicate or triplicate in a 96-well PCR plate. In each well, 10 µL of the sample were mixed with 15 µL of RT reaction cocktail containing RT buffer, MS2 RNA and primer B. The mixed samples were incubated at 37 °C for 90 min on a PCR thermocycler (Eppendorf MasterCycler ProS). Then, 25 µL of PCR cocktail containing PCR buffer, AmpliTaq Gold, TaqMS2 specific primers and a probe was added to each well and the reaction was run for 1 cycle at 95 °C for 10 min, 50 cycles at 95 °C for 15 s and 56 °C for 30 s on the QuantStudio 3 System (Thermo Fisher scientific, Waltham, MA, USA). The PERT assay was valid if the R^2^ > 0.97 for the HIV-1 RT enzyme standard curve and negative control was negative. The PERT activity in the samples were calculated based on the HIV-1 enzyme standard curve.

Inhibition of the Sf-RT activity was evaluated in the PERT assay by adding 10 μL of different concentrations of 3’-azido-2’,3’-dideoxythymidine-5’-triphosphate (AzTTP; TriLink Biotechnologies, San Diego, CA, USA; Catalogue number N-4009) to 90 μL of Sf9 supernatant, which was diluted at 1:10 with NZ/DTT buffer, and further diluted 1:10 prior to performing the PERT assay. HIV-1 RT enzyme was diluted to 1 × 10^6^ pU/mL with NZ/DTT buffer and similar concentrations of AzTTP were added to obtain the positive control. The final concentrations of AzTTP based on the 25 μL RT reaction mix were as follows: 2, 4, 20, and 40 μM. A sample without AzTTP (0 μM) was also included.

Ultracentrifugation and Gradient Ultracentrifugation. Sf9 filtered supernatant (800 µL) was ultracentrifuged at 124,406× *g* (45,000 rpm) for 1 h and for 20 h at 4 °C (Beckman TLA 45 rotor). After ultracentrifugation, the supernatant was removed, and the pelleted material was resuspended in the original volume (800 µL) of complete medium. Cell-free supernatants and resuspended pellets were stored in aliquots (10 µL) at −80 °C for analysis in the PERT assay.

For the OptiPrep gradient, 2 mL of 48%, 38%, 28%, 18% and 8% of OptiPrep density gradient medium (Millipore Sigma, Burlington, MA, USA; catalogue number D1556), diluted in PBS, were layered from bottom to top in an ultracentrifugation tube (Beckman Coulter, Indianapolis, IN, USA; catalogue number 344059). For sucrose gradient, 2 mL of 60%, 45%, 30%, 15% and 0% of sucrose (Millipore Sigma, catalogue number S0389) dilutions in PBS were layered from bottom to top in an ultracentrifugation tube. For both gradients, the tube with 10 mL of OptiPrep or sucrose was sealed with parafilm and tipped over gently to a horizontal position for 45 min. Then, the tube was switched to a vertical position gently and the seal was removed. The sample (0.5 mL) was added on top of the gradient. The tubes were ultracentrifuged at 122,000× *g* (31,400 rpm; SW41 rotor) for 16 h at 4 °C with the highest deceleration setting. Fractions (0.5 mL) were removed from the top to bottom of the gradient. Forty microliters of each fraction were used to measure the refractive index with a refractometer (Reichert Amterk, Depew, NY, USA) for calculating the density. Ten microliter aliquots from each fraction were stored at −80 °C for the PERT assays.

Size filtration. Sf9 supernatant (400 μL; 0.45 μm filter) was filtered using 0.2 μm, 300 K, 100 K, 30 K Nanosep centrifugal devices (Catalogue number: ODPTFE02C34, OD 300C33, OD100C33, OD030C33, respectively; Pall, Port Washington, NY, USA) and centrifuged at 12,000× *g* for 5–15 min until no fluid remained on top of the filter unit. The filtrates were collected and 10 μL was aliquoted for the PERT assay.

Transmission electron microscopy (TEM). Filtered supernatant from Sf9 cells was evaluated for particles by TEM as described previously [24]. Cyro-EM was performed by NanoImaging, Inc. (San Diego, CA, USA) using 3 μL of Sf9 cell supernatant that had been concentrated 30-fold by sequentially using centrifugal filters with 1,000,000- and 300,000-molecular-weight cutoffs (Pall Corporation, Port Washington, NY, USA). Complete medium was the negative control.

Thin sectioning of the pelleted virus from Sf9 cell supernatant was performed by SGS Vitrology (Glasgow, UK). The virus pellet was produced from a 10 mL sample by low-speed clarification (11,000× *g*, 10 min), filtration (pore size, 0.45 μm), and ultracentrifugation (150,000× *g* for 3 h). Sections were stained with 2% (w/v) ethanolic uranyl acetate and Reynolds’ lead citrate

Chemical induction studies. Chemical induction was performed according to our previously described strategy [17]. The optimum drug concentration was determined based on cell toxicity and cell recovery by treating Sf9 cells with different concentrations of 5-iodo-2′-deoxyuridine (IUdR Sigma, St. Louis, MO, USA, catalogue number 17125; stock solution of 75 mg per mL prepared in 1N NH4OH), 5-azacytidine (AzaC, Sigma, St. Louis, MO, USA, catalogue number A1287; stock solution of 1 mg/mL prepared in Grace’s Supplemented Insect Medium), sodium butyrate (NaB; Sigma, catalogue number B5887; stock solution of 0.9 M prepared in sterile water), and 12-O-tetradecanoyl-phorbol-13-acetate (TPA; Sigma, catalogue number P3766; stock solution, stock solution of 1 mg/mL prepared in dimethyl sulfoxide, DMSO).

Sf9 cells (1.6 × 10^6^; passage 23) were plated for 24 h in 25 cm^2^ flasks (to reach the beginning of the log phase based on the growth curve) before replacing the medium with medium containing different concentrations of AzaC (0, 1.25, 2.5, 5, 10, 20, 40 µg/mL), IUdR (0, 25, 50, 100, 200, 400 µg/mL), NaB (0, 0.25, 0.5, 1, 2, 4, 8, 16 mM) and TPA (0, 50, 100, 200, 400 ng/mL). After 48 h of drug treatment (equal to 1.25 times of the population doubling time of the cells) (designated as day 0), the cells were washed with medium three times and culturing continued with fresh complete medium. The culture was observed for cell toxicity and the medium was changed each day until the cells reached 95–100% confluence. The medium collected each day was filtered (0.45 μM filter; Corning, Corning, NY, USA catalogue number 430314) and 10 μL aliquots were stored at −80 °C for RT analysis using the PERT assay. Cells were set up in parallel without drug or with NH4OH (in the case of IUdR) and the medium collected was used as a negative control for the PERT assay.

Infectivity studies. The inocula used for infectivity studies were the cell-free supernatant collected from Sf9 cells (passage 20) and the cell-free supernatant collected from IUdR-treated Sf9 cells (200 μg/mL; Day 2), which had been filtered through a 0.45 μM filter (Corning, Corning, NY, USA, catalogue number 430314). SFV-1 (10 TCID_50_) was used as a positive control in an independent experiment to demonstrate the susceptibility of the target cell lines to retrovirus infection at 37 °C incubation. SFV-1 replication was based on progression of cytopathic effect (CPE) and increasing RT activity in PERT assay performed on selected samples [20,21].

The target cells were plated 24 h prior to infection to reach 70% confluence at the time of infection. The infectivity experiment was set up in 25 cm^2^ flasks by incubating A204, A549, MRC-5, Raji, and Vero cells with 2.5 mL of Sf9 supernatant, 2.5 mL of IUdR-treated Sf9 supernatant or 2.5 mL of Grace’s complete medium (as control), containing a final concentration of 4 µg/mL of polybrene (Millipore, Billerica, MA, USA, catalogue number TR-1003-G), at 28 °C for 2 h, before adding 2.5 mL of the target cells’ complete medium with polybrene, after which the cells were incubated for 24 h at 37 °C. The media from all of the cultures were replaced with target-cell medium after 3 rounds of washing and the cell lines were further incubated at 37 °C. The cells were passaged every 2–4 days upon reaching 95% confluence, at which time the medium was completely replaced with fresh medium. The cultures were regularly observed for CPE. All cultures without CPE were continued for at least 30 days. The cultures infected with SFV-1 were terminated upon reaching extensive CPE.

Filtered supernatants and washed cell pellets were collected and stored at −80 °C at every passage.

High-Throughput Sequencing analysis. DNA was extracted using a DNeasy Blood and Tissue Kit (Qiagen, Germantown, MD, USA; catalogue number 69504) from a pellet of MRC-5 cells collected on Day 2 after inoculation of the cells with IUdR-induced Sf9 cell supernatant. HTS whole-genome sequencing was performed by the CBER core facility using Illumina NovaSeq. The number of paired-end reads after trimming was 3,892,770,556 with an average read length of 146 bp. HTS data analysis was performed using CLC Genomics Workbench Version 23.0.2 (parameter length fraction = 0.3 or 0.5, similarity fraction = 0.9) to map the total reads against an in-house database of 571 retroelements identified in the Sf9 genome.

## 3. Results

### 3.1. Characterization of Extracellular Sf-RT Activity

**Figure 1:**
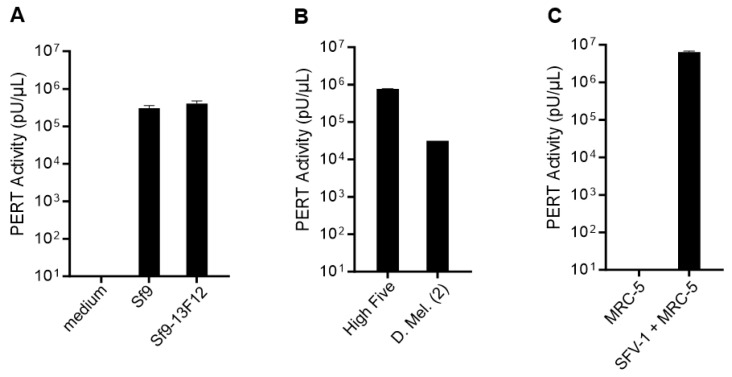
Quantitative analysis of RT activity in cell-free supernatant using the PERT assay. PERT activity is shown as pU/µL in the original test sample. (**A**) Sf9 and Sf9-13F12 insect cell lines including complete medium control; (**B**) High Five and D. Mel. (2) insect cell lines; and (**C**) uninfected and SFV-1 infected MRC-5 cells. Negative RT result indicates below limit of detection of the PERT assay (<10 pU of HIV-1 RT enzyme). Results shown in each panel are the mean ± SD of duplicate or triplicate samples tested in the same PERT assay.

The Sf9 cell line can be cultured adherent or in suspension, with or without a serum. In this study, the cells were grown as an adherent culture with a serum. The presence of RT activity in cell-free supernatant of Sf9 cells was evaluated using the highly sensitive PERT assay. The results shown in Figure 1A indicated that a similar RT activity (about 10^5^ to 10^6^ pU/µL) was produced by Sf9 cells, which are infected with the Sf-rhabdovirus, and Sf9-13F12 cells, which are Sf-rhabdovirus-free cells [25,28]. No RT activity was detected in the complete medium. A similar level of RT activity was also detected in the cell free supernatant from insect High Five cells and a lower level (about 10^4^ to 10^5^ pU/µL) was detected from D. Mel. (2) cells (Figure 1B). A high level of RT activity (about 10^6^ to 10^7^ pU/µL) was detected in the cell-free supernatant of SFV-1 infected MRC-5 cells, which represented a positive retrovirus control for the PERT assay. No RT activity was detected from the uninfected MRC-5 cells (Figure 1C).

**Figure 2:**
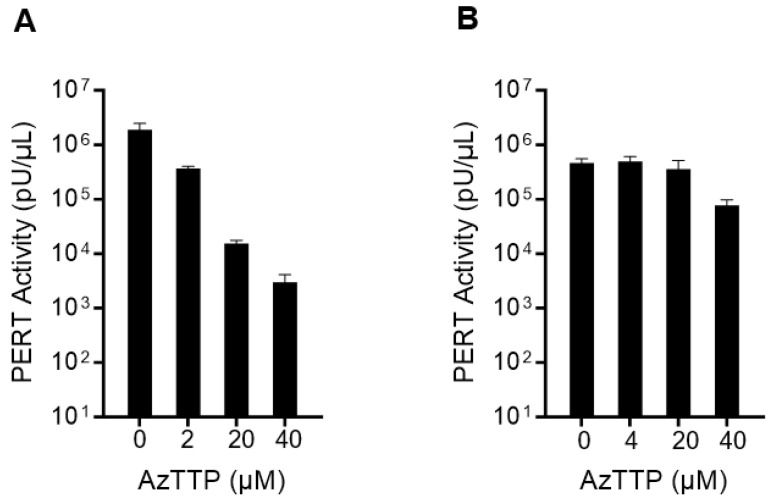
PERT analysis of AzTTP inhibition of retroviral RT activity. AzTTP was added at final concentrations of 0, 2 or 4, 20, and 40 µM in the RT step of the PERT assay. (**A**) HIV-1 RT enzyme; and (**B**) Sf9 RT in filtered supernatant.

Since the PERT assay can detect retroviral RT and some cellular DNA polymerases, the specificity of the Sf-RT activity, being of retroviral origin, was determined using AzTTP, a known inhibitor of HIV-1 RT and DNA polymerase gamma [29]. In Figure 2, the HIV-1 RT enzyme was diluted to 10^6^ pU/µL, comparable to the RT activity in the Sf9 supernatant. The results showed that the HIV-1 RT activity decreased significantly with an increasing concentration of AzTTP: a 633-fold decrease was seen with 40 µM of AzTTP. However, the Sf-RT activity remained the same up to 20 µM of AzTTP and decreased 6-fold at 40 µM of AzTTP. These results indicated that the RT in the Sf9 supernatant is retroviral-related and distinct from the HIV-1 RT.

**Figure 3:**
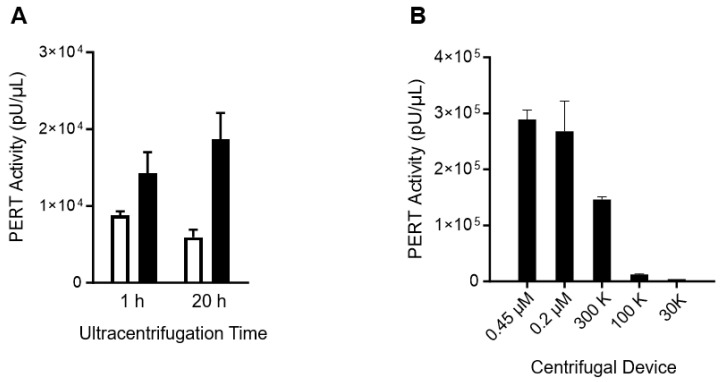
Analysis of particle-associated RT activity. (**A**) Sf9 filtered supernatant was ultracentrifuged at 45,000 rpm for 1 h and 20 h. The PERT results of the resuspended pellet (black bar) and the non-pelleted material (white bar) are shown. (**B**) Sf9 filtered supernatant (0.45 µm) was the starting sample placed in the upper chamber of each 0.2 µm, 300 K, 100 K and 30 K centrifugal device. Filtrate was collected after spinning at 14,000 rpm (Section 2) and analyzed in the PERT assay. Results of the mean ± SD of duplicate samples tested in the same PERT assay are shown.

To determine if the RT was particle-associated, Sf9 cell-free supernatant was ultracentrifuged at 45,000 rpm (124,406× g) for 1 h and for 20 h. The results shown in Figure 3A demonstrate that about 30% more RT activity was pelleted when the duration of ultracentrifugation was increased from 1 h to 20 h. Compared to enveloped retroviruses like HIV-1, which can be pelleted by ultracentrifugation at 25,500× *g* for 1 h [30], it seems that the Sf9 supernatant contained various sizes of particles and some RT-particles needed an extended ultracentrifugation time to be pelleted. It was notable that some of the RT activity still remained in the supernatant after 20 h of ultracentrifugation. To characterize the particle size associated with the Sf9 RT activity, cell-free supernatant was filtered through centrifugal devices with different membrane pore sizes. Figure 3B shows that the filtrate contained a similar level of RT activity using 0.45 and 0.2 µm filters whereas about half of the RT activity passed through the 300 K device, which had a 35 nm membrane nominal pore size for 90–200 nm viruses or particles (based on the manufacturer’s instruction), and about 5% of the RT activity passed through the 100 K device, whch had a 10 nm membrane nominal pore size for 30–90 nm viruses or particles. No RT activity was detected in the filtrate of the 30 K device. These results further demonstrated that particles produced from Sf9 cells were a mix of different sizes.

**Figure 4:**
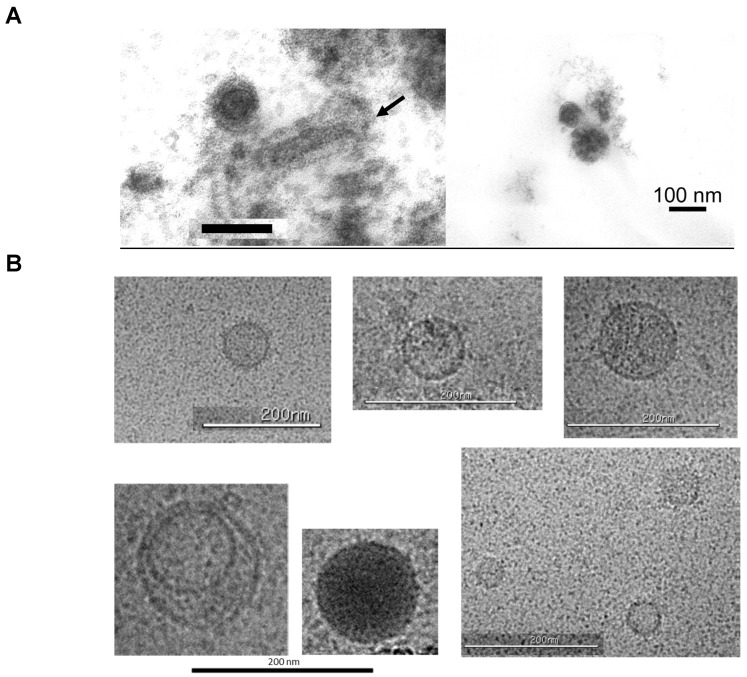
Electron microscopy of viral-like particles in Sf9 supernatant. (**A**) Thin section of pelleted virus from Sf9 cell supernatant (SGS Vitrology). Arrow indicates Sf-rhabdovirus [24]. Bar is shown for 100 nm. (**B**) Cryo-EM images (NanoImaging, Inc.). Bar is indicated for 200 nm.

TEM analysis indicated that some spherical retrovirus-like particles, about 65–110 nm in diameter, were seen in thin-sections that were prepared from the ultracentrifuged pellet of Sf9 supernatant (Figure 4A). Some resembled the 50 nm type A retroviral-like particles previously described to be present in *D. melanogaster* cells [13]. The bacilliform Sf-rhabdovirus of about 170 to 190 nm in length, which was described previously [24], was also observed in the same image (Figure 4A, left panel, indicated by the arrow). Cryo-EM showed 40–120 nm unilamellar spherical particles that appeared to have a textured appearance and proteinaceous material on their surface (Figure 4B). Additionally, a few spherical particles contained highly electron-dense structures (Figure 4B, lower middle panel) and some were present inside larger vesicles (Figure 4B, lower left panel). Rod-like Sf-rhabdovirus with a scaled appearance, which has been described previously, was present in the same sample (Figure 1F; [24]). The results from the EM analysis confirmed the heterogeneity in the size of the RT-particles produced extracellularly from Sf9 cells, which was also found using the Nanosep centrifugal devices (results shown in Figure 3B). The extracellular particles seen by electron microscopy indicated a mix of particles, including retroviral-like and possibly extracellular vesicles and exomes [31].

**Figure 5:**
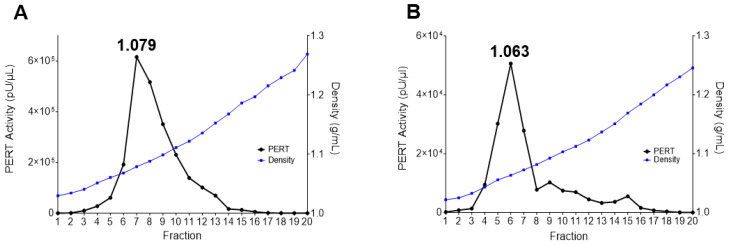
Optiprep density gradient analysis of RT activity in insect cell supernatant. Cell supernatant (0.5 mL) from Sf9 cells (**A**) or D. Mel. (2) cells (**B**) were added on the top of an OptiPrep gradient and the tubes were ultracentrifuged at 122,000× *g* for 16 h. Fractions (0.5 mL) were removed from the top to bottom of the gradient. Ten microliters from each fraction were analyzed for RT activity by the PERT assay (left *Y*-axis). Forty microliters from each fraction were analyzed for density by a refractometer (right *Y*-axis). The density at the peak RT activity is indicated.

The buoyant density of the Sf-RT was analyzed on an OptiPrep gradient and the results are shown in Figure 5A. The peak RT corresponded to a low density of about 1.08 g/mL (fraction 7). Interestingly, a similar low density RT peak was seen upon analyzing the cell-free supernatant from D. Mel. (2) cells (fraction 6; Figure 5B). This was also noticed in the gradient analysis of D. Mel. (2) cell lysate along with an RT peak at 1.22 g/mL, corresponding to intracellular A-type particles [13]. The results indicated that the insect cells express RT particles that have a unique low buoyant density that is distinct from the 1.16 g/mL density expected from known enveloped retroviruses [32].

### 3.2. Characterization of Inducible, Endogenous RT from Sf9 Cells

**Figure 6:**
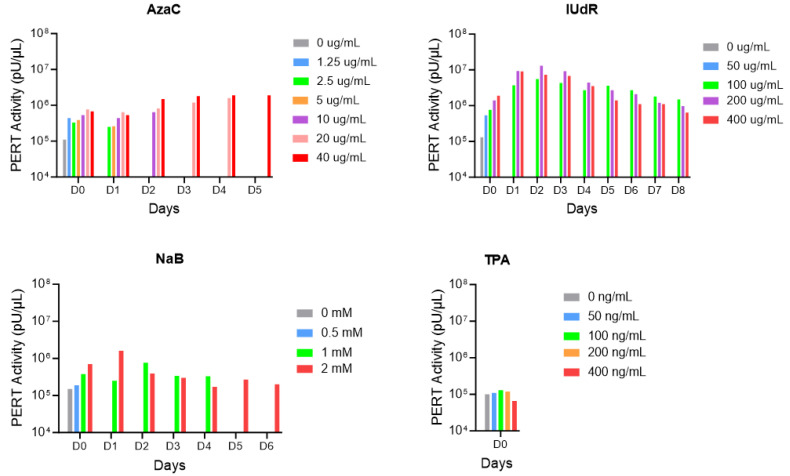
PERT analysis of chemically-induced RT activity from Sf9 cells. Sf9 cells were treated for 48 h with a range of drug concentrations [IUdR, 0–400 μg/mL; AzaC, 0–40 μg/mL; NaB, 0–16 mM (data for 4–16 mM not shown due to high cell toxicity); and TPA, 0–400 ng/mL]. Day 0 is the day of drug removal. Ten microliters of filtered supernatants were collected every day from drug-treated cells and analyzed by the PERT assay.

A broad range of drug concentrations were evaluated for effective induction of RT activity from Sf9 cells with AzaC, IUdR, NaB and TPA, based on our previous chemical induction studies [17]. Figure 6 shows the PERT results for the treatment of Sf9 cells with different drug concentrations at the indicated times until 95–100% confluence was reached, which varied based on cell recovery due to drug toxicity. In the case of TPA, there was no drug toxicity even at the highest concentration and the cells reached confluence on D0 (equal to 48 h after drug treatment). The highest RT activity was induced with 200 µg/mL of IUdR on D2, albeit with significant cell toxicity (less than 20% cell confluence). Compared to the untreated Sf9 cells (0 µg/mL on D0), the peak RT activity increased 33-fold when treated with IUdR. A low peak of RT activity was induced by 40 µg/mL of AzaC on D4 and by 2 mM NaB on D1. NaB had a high toxicity for concentrations above 2 mM.

**Figure 7:**
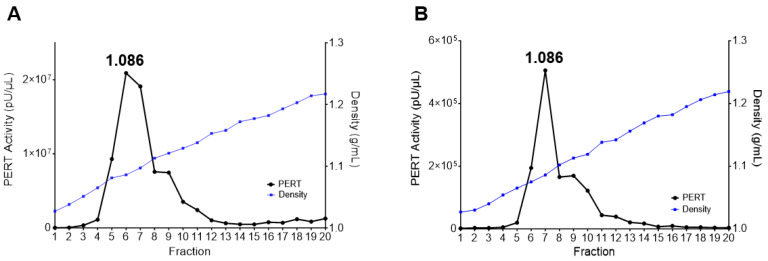
Sucrose gradient density analysis of supernatant from drug-treated and untreated Sf9 cells. Cell-free supernatant (0.5 mL) from (**A**) Sf9 cells treated with IUdR (day 2, 200 μg/mL) and (**B**) untreated Sf9 cells was added on top of a sucrose gradient and the tubes were ultracentrifuged at 122,000× *g* for 16 h. Fractions (0.5 mL) were removed from the top to bottom of the gradient. Ten microliters from each fraction were analyzed for RT activity by the PERT assay (left *Y*-axis). Forty microliters from each fraction were analyzed for density by a refractometer (right *Y*-axis). The density at the peak RT activity is indicated.

Sucrose gradient analysis of filtered supernatant of the D2-RT activity induced by the treatment of Sf9 cells with IUdR (200 μg/mL) indicated that the majority of the RT activity corresponded to the same buoyant density of 1.086 g/mL (fraction 6) as seen for the untreated Sf9 cells (fraction 7) (Figure 7A and Figure 7B, respectively). It should be noted that the density of enveloped retroviruses and non-enveloped A-type retrovirus particles would correspond to a density of 1.16 g/mL (fraction 13) and 1.22 g/mL (fraction 20), respectively.

### 3.3. Infectivity Analysis of Sf-RT Particles

**Figure 8:**
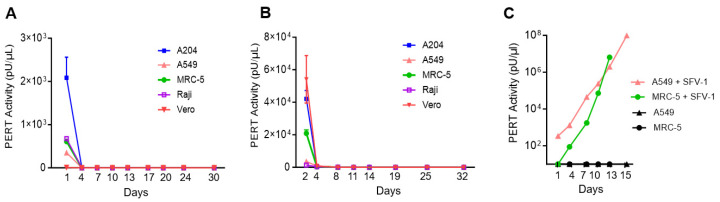
Infectivity analysis of Sf-RT particles. A204 cells, A549 cells, MRC-5 cells, Raji cells and Vero cells were inoculated with cell-free supernatant from (**A**) untreated Sf9 cells and (**B**) D2 (200 μg/mL) of IUdR-treated Sf9 cells. Filtered supernatant was collected from the inoculated cells at the first cell passage post-inoculation until its termination at day 30 or 32. (**C**) SFV-1 infected cells are shown as a positive control for retrovirus infection of A549 and MRC-5 cells; uninoculated cells are included as control. Samples were analyzed using the PERT assay.

To evaluate whether the extracellular RT-particles in the supernatants of Sf9 cells and IUdR-induced Sf9 cells was associated with replication-competent, supernatants with peak RT activity were used to inoculate cell lines known to be susceptible to retrovirus infection [20,21]. The PERT results demonstrated the detection of only the input RT activity in the supernatants of Sf9 cells and IUdR-treated cells (Figure 8A and Figure 8B, respectively), which gradually reduced to background or undetectable levels, without any amplification during more than 30 days of culture. No RT activity was detected in the uninoculated target cells. In contrast, an increasing RT activity was seen in cells infected with the SFV-1 retrovirus (panel C). These results indicated the absence of an infectious retrovirus produced from Sf9 cells that could replicate in the target cell lines.

Since Sf9 cells were cultured at 28 °C, which is the temperature for culturing insect cell lines, we evaluated the effect of temperature on the RT activity in additional infectivity studies. MRC-5 and Vero cells were incubated for 2 h at 28 °C with fresh, cell-free supernatant from Sf9 cells (without freeze–thaw), followed by further culturing at both 37 °C and 28 °C. The cells failed to grow at 28 °C, without an increase in RT. No increase in RT activity was seen in the cells cultured at 37 °C.

## 4. Discussion

The safety of biological products is assured by demonstrating the absence of adventitious viruses in the final product. This includes extensive testing at various steps during the manufacturing process, and involves testing starting materials such as cell lines [33,34]. Some cell lines used for the production of biologics are known to produce retroviral particles. In such cases, it is expected that the manufacturing process demonstrates the clearance of the viral particles (as in the case of Chinese hamster ovary CHO cells) [35] or the extensive analysis of the particles for evaluating its potential risk for human infection based on physical and genomic characterization and infectivity studies (as in the case of chicken embryo fibroblast cultures) [20,36,37,38]. Some insect cell lines have been used for manufacturing of biological products [39], but the analysis of retroviral particles have only been performed for *Drosophila* [13,15,16]. A highly sensitive PCR-based RT assay was used to detect the presence of RT activity expressed from Sf9 cells, which are used for baculovirus-expressed products [6]. However, a systematic characterization was not performed to determine if the RT activity was associated with retroviral particles, whether the associated viral genome was complete and could encode proteins from putative retroviral genes, or if the particles were infectious and posed a potential safety concern for humans. This paper describes the evaluation of the physical and infectious properties associated with Sf-RT activity. Further, investigations of the potential endogenous retroviral sequences encoding the Sf-RT are ongoing.

Using the PERT assay, we found that the Sf9 cell supernatant contained about 10^5^ pU/μL of RT activity, which could be partially pelleted by ultracentrifugation. TEM analysis of cell-free supernatant revealed the presence of heterogenous particles with varying sizes and structures, some of which resembled type A retroviral-like particles that have been previously described in *Drosophila* tissues and cell lines [13,14,16,40]. It should be noted that the extracellular viral-like particles produced from Sf9 cells corresponded to a low buoyant density RT, which peaked at about 1.08 g/mL, which was also seen in the previous study with *D. melanogaster* cell lysate but was not followed up [13]. Additionally, the Sf-RT activity was distributed broadly around the peak RT in the density gradient analysis, suggesting the association with particles of different sizes and types. This was evident by electron microscopy studies which showed retrovirus-like particles that could possibly be associated with low-density lipoproteins while egressing from the cells or the particles may have interacted with “light” extracellular vesicles, resulting in the low buoyant density [41]. Interestingly, the hepatitis C virus has been shown to have a low buoyant density by associating with very low-density lipoproteins, whereas the naked viruses are of high density [42,43,44]. Our ongoing studies using various membrane dissociation and disruption reagents and qPCR assays based on an HTS analysis of the Sf9 genome [45], transcriptome, and virome will help provide further insights into the structure and genomes of the Sf-RT particles. Our initial results indicate the presence of different insect retrotransposons expressed from the Sf9 cell line, for example Ty3-, Ty1-, and Bel/Pao-related retrotransposons [9].

To further investigate the presence of endogenous retroviruses or other latent viruses that can encode particles in Sf9 cells, we used four chemical inducers with different mechanisms of virus activation [17,18,19,46,47,48,49], including IUdR, which is generally used to induce endogenous retroviruses. The results of our study demonstrated that the IUdR treatment resulted in a higher RT activity from the Sf9 cells, compared with AzaC and NaB, whereas TPA induction did not significantly affect the RT activity. The induction of a high RT activity from Sf9 cells suggests that it is produced from endogenous retroviruses or retrotransposons. Interestingly, the peak RT activity had a low density compared with the expected density of known retroviruses [32]. We have further investigations ongoing to characterize the novel RT-viral particles associated with a low buoyant density.

The gypsy retrotransposon in *D. melanogaster* was reported to be infectious and transmitted horizontally and vertically in its host [40,50], and also to be propagated in *D. hydei* cells [51]. To address the potential safety concerns related to RT particles in the Sf9 cell line, we performed infectivity studies to investigate their potential replication in mammalian cell lines. The results demonstrated that the Sf-RT particles expressed from Sf9 cells or after induction by chemical treatment were not replication competent in the target cell lines used in the study. We further showed that mammalian cells cannot support the replication of the Sf-RT particles at the 28 °C temperature that may be important for their production in the Sf9 cells. Other insect cell lines that were cultured at 28 °C, such as the High Five cell line (BTI-Tn-5B1-4) and D. Mel. (2), could be potential cell lines for evaluating the infectivity of Sf-RT particles, but they also produce RT activity, which would interfere in interpreting the results of such infectivity studies. Therefore, we have focused our efforts on characterizing the viral genomes in the Sf-RT particles and focus on those with putative ORFs for developing virus-specific PCR assays to investigate their potential entry/integration in mammalian cells.

## Data Availability

Data can be provided upon request.
